# 596. The ID Physician Is Out: Are Remote ID E-Consults an Effective Substitute?

**DOI:** 10.1093/ofid/ofab466.794

**Published:** 2021-12-04

**Authors:** Sui Kwong Li, Carolyn Fernandes, Sowmya Nanjappa, Sarah Burgdorf, Vidya Jagadeesan, Bettina Knoll, Shanza Khan, Nupur Gupta, John Mellors, Rima Abdel-Massih, Rima Abdel-Massih

**Affiliations:** 1 University of Pittsburgh Medical Center, Pittsburgh, Pennsylvania; 2 University of Pittsburgh, Pittsburgh, Pennsylvania

## Abstract

**Background:**

Telemedicine (TM) can provide specialty ID care for remote and underserved areas; however, the need for dedicated audio-visual equipment, secure and stable internet connectivity, and local staff to assist with the consultation has limited wider implementation of synchronous TM. ID e-consults (ID electronic consultations or asynchronous™) are an alternative but data are limited on their effectiveness, especially patient outcomes.

**Methods:**

In the setting of the COVID-19 pandemic and ID physician outage, we were asked to perform ID e-consults at a 380-bed tertiary care hospital located in Blair County, PA. We performed retrospective chart reviews of 121 patients initially evaluated by ID e-consults between April 2020 and July 2020. Follow-up visits were also conducted via e-consults with or without direct phone calls with the patient. Key patient outcomes assessed were length of stay (LOS), disposition after hospitalization, 30-day mortality from initial ID e-consult and 30-day readmission post-discharge.

**Results:**

The majority of patients were white males and non-ICU (Table 1). The most common ID diagnosis was bacteremia (27.3%, 33/121), followed by skin and soft tissue infections (15.7%, 19/121) and bone/joint infections (14.9%, 18/121) (Figure 1). Table 2 shows patient outcomes. Average total LOS was 11 days and 7 days post-initial ID e-consult. 48.7% (59/121) of patients were discharged home and 37.2% (45/121) to a post-acute rehabilitation facility. 2.5% (3/121) of patients required transfer to a higher level of care facility; none of which were to obtain in-person ID care. The index mortality rate was 3.3% (4/121), which appears to be lower than published data for in-person ID care. The 30-day mortality rate was 4.1% (5/121), which is also comparable to previously reported for ID e-consults. 25.6% (31/121) of patients required readmission within 30 days but only 14.0% (17/121) were related to the initial infection.

Table 1. Demographics

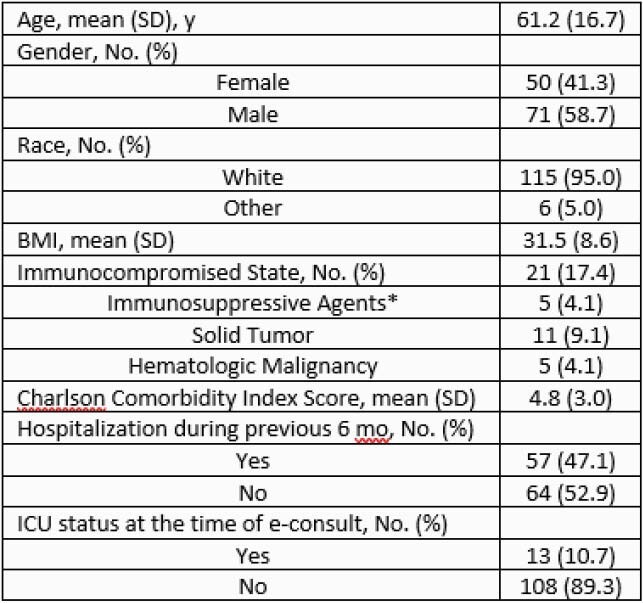

*Immunosuppressive agents include: Apremilast, Dasatinib, Etanercept, Remicade, Rituximab, and Prednisone >10 mg/day

Figure 1. Variety of ID Diagnoses made by e-consults

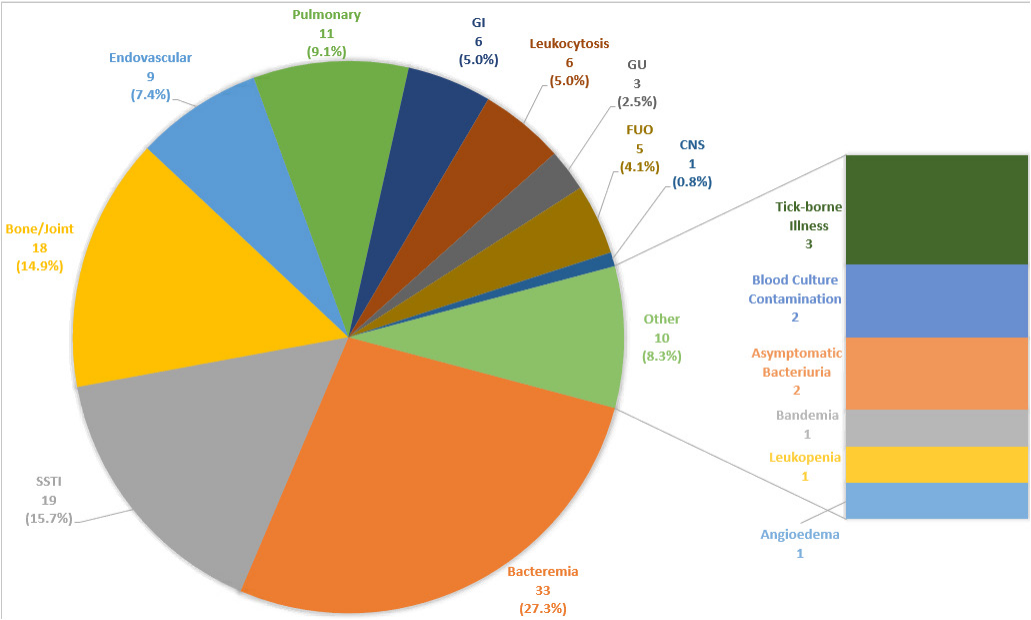

Table 2. Outcomes

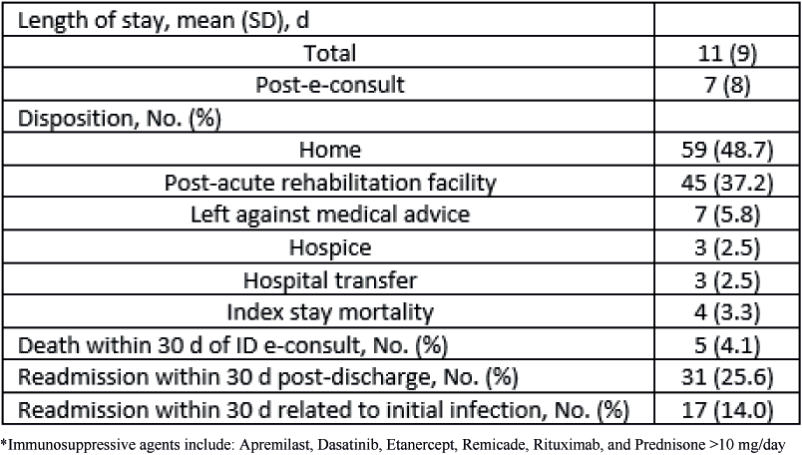

**Conclusion:**

We believe that this is the first report of the implementation of ID e-consults at a tertiary care hospital. Mortality rates appear to be comparable to in-person ID care. In the absence of in-person ID physicians, ID e-consults can be a reasonable substitute. Further study is required to compare performance of ID e-consults to in-person ID consults.

**Disclosures:**

**John Mellors, MD**, **Abound Bio, Inc.** (Shareholder)**Accelevir** (Consultant)**Co-Crystal Pharma, Inc.** (Other Financial or Material Support, Share Options)**Gilead Sciences, Inc.** (Advisor or Review Panel member, Research Grant or Support)**Infectious DIseases Connect** (Other Financial or Material Support, Share Options)**Janssen** (Consultant)**Merck** (Consultant) **Rima Abdel-Massih, MD**, **Infectious Disease Connect** (Employee, Director of Clinical Operations) **Rima Abdel-Massih, MD**, Infectious Disease Connect (Individual(s) Involved: Self): Chief Medical Officer, Other Financial or Material Support, Other Financial or Material Support, Shareholder

